# Intervening in the local health system to improve diabetes care: lessons from a health service experiment in a poor urban neighborhood in India

**DOI:** 10.3402/gha.v8.28762

**Published:** 2015-11-16

**Authors:** Upendra Bhojani, Patrick Kolsteren, Bart Criel, Stefaan De Henauw, Thriveni S. Beerenahally, Roos Verstraeten, Narayanan Devadasan

**Affiliations:** 1 Institute of Public Health, Bangalore, India; 2 Department of Public Health, Institute of Tropical Medicine, Antwerp, Belgium; 3 Department of Public Health, Ghent University, Ghent, Belgium

**Keywords:** diabetes, health system, healthcare services, poverty, health education, medication, standard treatment guidelines, RE-AIM framework, India

## Abstract

**Background:**

Many efficacious health service interventions to improve diabetes care are known. However, there is little evidence on whether such interventions are effective while delivered in real-world resource-constrained settings.

**Objective:**

To evaluate an intervention aimed at improving diabetes care using the RE-AIM (reach, efficacy/effectiveness, adoption, implementation, and maintenance) framework.

**Design:**

A quasi-experimental study was conducted in a poor urban neighborhood in South India. Four health facilities delivered the intervention (*n*=163 diabetes patients) and the four matched facilities served as control (*n*=154). The intervention included provision of culturally appropriate education to diabetes patients, use of generic medications, and standard treatment guidelines for diabetes management. Patients were surveyed before and after the 6-month intervention period. We did field observations and interviews with the doctors at the intervention facilities. Quantitative data were used to assess the reach of the intervention and its effectiveness on patients’ knowledge, practice, healthcare expenditure, and glycemic control through a difference-in-differences analysis. Qualitative data were analyzed thematically to understand adoption, implementation, and maintenance of the intervention.

**Results:**

Reach: Of those who visited intervention facilities, 52.3% were exposed to the education component and only 7.2% were prescribed generic medications. The doctors rarely used the standard treatment guidelines for diabetes management. Effectiveness: The intervention did not have a statistically and clinically significant impact on the knowledge, healthcare expenditure, or glycemic control of the patients, with marginal reduction in their practice score. Adoption: All the facilities adopted the education component, while all but one facility adopted the prescription of generic medications. Implementation: There was poor implementation of the intervention, particularly with regard to the use of generic medications and the standard treatment guidelines. Doctors’ concerns about the efficacy, quality, availability, and acceptability by patients of generic medications explained limited prescriptions of generic medications. The patients’ perception that ailments should be treated through medications limited the use of non-medical management by the doctors in early stages of diabetes. The other reason for the limited use of the standard treatment guidelines was that these doctors mainly provided follow-up care to patients who were previously put on a given treatment plan by specialists. Maintenance: The intervention facilities continued using posters and television monitors for health education after the intervention period. The use of generic medications and standard treatment guidelines for diabetes management remained very limited.

**Conclusions:**

Implementing efficacious health service intervention in a real-world resource-constrained setting is challenging and may not prove effective in improving patient outcomes. Interventions need to consider patients’ and healthcare providers’ experiences and perceptions and how macro-level policies translate into practice within local health systems.

India is a home to over 68.8 million people with diabetes, a majority of whom struggle to get quality healthcare (1). The need to reorient and strengthen the existing health systems to provide effective response to the care demands of people with chronic conditions, such as diabetes, is globally recognized (2). In order to address the rising burden of diabetes, India launched pilots of a national program in 2008 that, among other things, aimed at reorienting the healthcare delivery system (3). Our earlier study in a poor urban neighborhood in South India, however, revealed many gaps in the organization of diabetes care in the local health system (4). To tackle these gaps, healthcare providers in the neighborhood were consulted to identify their preferred interventions to improve diabetes care (5–11). These included, for example, the provision of culturally appropriate health information as well as the use of standard treatment guidelines and low-cost generic medications for diabetes management.

Systematic reviews of randomized controlled trials showed that these suggested interventions are likely to be efficacious. Health education to diabetes patients has been shown to improve patients’ knowledge and glycemic control (5–15). Interventions targeted at patients, prior to the consultation, using coaching, checklists, or decision aids (video, pamphlets) can enhance the patients’ knowledge about treatment choices, their self-efficacy, their participation in decision-making, and patient–provider communication (16, 17). Finally, interventions targeting primary care providers, such as the use of printed educational materials or personalized outreach educational visits, have been shown to enhance professional practices of healthcare providers, including drug prescribing patterns (18, 19).

There is, however, a dearth of studies on the application of these interventions in real-world settings, especially in the local health systems of low- and middle-income countries. Sanders and Haines (20) emphasize the usefulness of health system research, especially implementation research. There remains a gap between the available knowledge and translating it into practice within health systems (20). More recently, prominent global actors echoed this need (21). Glasgow et al. (22), in response to the dominant ‘efficacy’ paradigm of the randomized controlled trials, developed the RE-AIM framework to analyze five important aspects of public health interventions (reach, efficacy/effectiveness, adoption, implementation, and maintenance). Using the RE-AIM framework, we aimed to evaluate a health service intervention to improve diabetes care in the local health system of a poor urban neighborhood.

## Methods

### Ethics statement

The Institutional Ethics Committee at the Institute of Public Health (India), the Institutional Review Board at the Institute of Tropical Medicine (Belgium) and the Ethics Committee at the University of Antwerp (Belgium) approved this study. The study was explained to the diabetes patients before informed written consent was sought, separately for the surveys and blood sugar tests. The study was explained to the doctors at the intervention health facilities before informed written consent was sought, separately for being part of the study (and agreeing to implement the intervention) and for the interviews at the end of the intervention.

### Study setting

We conducted the study in Kadugondanahalli (KG Halli), one of the administrative units of Bangalore. Bangalore is a metropolitan city, capital of the southern Indian state of Karnataka. KG Halli is a poor neighborhood housing a slum and a population of around 45,000 in an area of less than one square kilometer. It has two government health centers, one providing limited primary care and the other providing primary care and some specialist care. It has over 32 private health facilities: Four are hospitals providing primary, specialist, and inpatient care, while the rest are single-doctor clinics providing primary care on an outpatient basis. Private facilities provide care on a fee-for-service basis. Government centers provide free care for the people living in below-the-poverty-line households and charge nominal fees to other patients. For more details on chronic conditions and health system organization in KG Halli, please refer to our earlier work (4, 23–25).

### Intervention design and components

We conducted a quasi-experimental study with an intervention and a control group. The Institute of Public Health (Bangalore) has been implementing the Urban Health Action Research Project in KG Halli since 2009 with an aim to improve the quality of healthcare for its residents by working with residents, the healthcare providers, and the health authorities. As part of this project, six rounds of dialogue with local healthcare providers were organized between August 2011 and March 2012 to discuss residents’ health issues, including chronic conditions and the potential solutions (5–10). Subsequently, in July 2013, individual meetings were held with doctors – healthcare providers, irrespective of their training background, who serve in clinic or hospitals and are generally the first point of consultation for diabetes patients – to share the findings of an earlier study (4) on gaps in the organization of diabetes care and to seek their suggestions on improving diabetes care (11). Based on the obtained suggestions and literature, we identified three intervention components: 1) provision of culturally appropriate diabetes education; 2) prescription of generic medications; and 3) use of standard treatment guidelines (STG) for diabetes management. Table 1 provides details on the intervention that was delivered at the four intervention health facilities over a 6-month intervention period. The control health facilities followed the regular health service provision.

**Table 1:** Intervention components and strategies

Intervention component	Intervention strategy	Content/support material	Primary target population
Provision of culturally appropriate diabetes education.	Display of three posters at conspicuous places within the patient waiting area at the intervention sites.	Posters contained contextually relevant images with minimal text in three commonly spoken languages (Urdu, Kannada, and Tamil), apart from English. They covered different aspects of diabetes education: 1) ‘Symptoms of sugar becoming high or low in blood’ (about diabetes and hyper- and hypoglycemia); 2) ‘Self-care for diabetes’ (about diet, tobacco consumption, and regular medication); and 3) ‘Self-care for diabetes’ (about indoor and outdoor exercise and foot care).	Diabetes patients
	Installation of television monitors to broadcast seven videos at conspicuous place within the patient waiting area at intervention sites.	The monitors displayed, sequentially, three videos in Kannada^a^, three videos in Urdu^b^, and one video in Tamil^c^. These videos ranged from 56 s to 15 min in length and covered issues related to diabetes and its management.	Diabetes patients
Use of generic medications for diabetes management.	Doctors at the intervention sites should prescribe generic medications, to the extent possible, for diabetes management.	Doctors were provided with a list of generic medications made available within KG Halli at low cost to the patients.	Doctors at the intervention sites
Use of standard treatment guidelines for diabetes management.	Doctors at the intervention sites to follow, to the extent possible, the standard treatment guidelines for diabetes management.	One-on-one meetings lasting from 30 to 45 min with doctors at the intervention sites to hand over copies of and discuss the standard treatment guidelines^d^ for diabetes management along with the fact sheets highlighting important aspects of the guidelines.	Doctors at the intervention sites

a Two of the Kannada videos were developed by the Swami Vivekananda Youth Movement, a nongovernmental organization, for use in primary care settings and included 1) *Diabetes II*, available from www.youtube.com/watch?v_W3ayZCV3R0U; and 2) *Food Habits in Diabetes*, available from www.youtube.com/watch?v_33eXNFUV0fE. The third Kannada video was developed by HealthBox India Trust, a non-governmental organization based in Karnataka.

b All the three Videos were developed by HeartFile, a non-governmental organization based in Pakistan, using imagery and a dialect similar to those in KG Halli, and included 1) *Diabetes – The Decision Is Yours*, available from www.youtube.com/watch?v_EGiH15R5Z_o; 2) *Diabetes – Two Stories*, available from www.youtube.com/watch?v_f4nY42kfBX8; and 3) *Diabetes – A Few Important Points*, available from www.youtube.com/watch?v_GM7_x0T8-Yc.

c Tamil video was developed by HealthBox India Trust, a non-governmental organization based in Karnataka.

d Guidelines for management of type-2 diabetes developed by the Indian Council of Medical Research (2005), available from www.icmr.nic.in/guidelines_diabetes/guide_diabetes.htm.

### Sampling framework

#### Sampling of health facilities

Based on willingness of the doctors to adopt the intervention in their routine practice, we conveniently sampled seven health facilities (one government, six private) from KG Halli and assigned them to the intervention group. The government facility had a mandate to provide free care and as such no control facility could be matched. Two of the six private facilities were technically unable to implement certain aspects of the intervention and were excluded. Hereafter, we will refer to the remaining four facilities as *intervention sites* and the doctors delivering care at these facilities as *intervention doctors*. Next, four control health facilities similar to the intervention sites in terms of type of clinic/hospital, level of formal training of doctors, and the usual load of diabetes patients seeking care from these facilities were matched. Table 2 provides details on intervention and control sites including doctors’ educational background and the services available at these sites.

**Table 2:** Characteristics of the intervention and control sites

Respondent number	Study sites	Delivery platform and services	Training of GP/respondent	Other remarks
R1	Intervention site 1	Clinic providing primary and limited specialist care on an outpatient basis	Graduated in modern medicine, with a fellowship in diabetology; practicing modern medicine	
	Control site 1	Clinic providing primary care on an outpatient basis	Graduated in modern medicine; practicing modern medicine	
R2	Intervention site 2	Clinic providing primary care on an outpatient basis	Graduated in Ayurveda with a course in integrated medicine; practicing modern medicine	
	Control site 2	Clinic providing primary care on an outpatient basis	Graduated in Unani; practicing modern medicine	
R3	Intervention site 3	Hospital with a few inpatient beds, a pharmacy, and a laboratory; provides primary care	Graduated in modern medicine; practicing modern medicine	Presence of other doctor(s) trained in Ayurveda and practicing modern medicine
	Control site 3	Clinic with a few inpatient beds (only for day admissions) and a laboratory; provides primary care	Graduated in modern medicine; practicing modern medicine	
R4	Intervention site 4	Hospital with inpatient beds, a pharmacy and a laboratory; providing primary and specialist care	Graduated in modern medicine; practicing modern medicine	Presence of other doctor(s) trained in Ayurveda/Unani and practicing modern medicine; specialists available for a limited time of the day
	Control site 4	Hospital with inpatient beds, a pharmacy, and a laboratory; provides primary and specialist care	Graduated in modern medicine; practicing modern medicine	Presence of other doctor(s) trained in Ayurveda/Unani and practicing modern medicine; specialists available for a limited time of the day

#### Sampling of patients

When estimating the minimum sample size for the study, we expected certain improvements in the effectiveness measures. These primary measures and the change expected in them included the following: 1) a two-point increase in the patient knowledge score (range 0–15); 2) a five-point decrease in the mean blood sugar level; and 3) a 50% reduction in the mean out-of-pocket expenditure. Considering a significance level of 0.05, 80% power, and average values of patient knowledge score, random blood sugar, and out-of-pocket expenditure from earlier studies (23, 26–28), we estimated the required minimum sample size to be 69 patients per group. We used STATA (StataCorp LP 11.2) to calculate the sample size and took the highest number needed for one of the three indicators. To accommodate for possible correlations across the groups, loss to follow-up and divergences in health-seeking behavior leading to crossover (i.e. patients from the control group visiting intervention sites), and lack of visits to study sites, we added another 100, making the final target sample size 169 patients per group.

We sampled these patients with help from community health workers as part of the Urban Health Action Research Project. These health workers were women from KG Halli and surrounding neighborhoods with a socioeconomic background similar to that of the people in KG Halli. They had been working in KG Halli for around 4 years making routine visits to households in their respective areas. They approached diabetes patients at their residence. People between the ages of 15 and 64 years, residing within KG Halli, with self-reported diabetes mellitus type 2, and who sought care from one of the intervention or control sites at least once in the last 3 months were considered eligible for the study. Severely ill or mentally incompetent patients were excluded.

### Measures, data collection tools, and analysis

Table 3 provides the measures and data collection tools used in the study to assess various aspects of the intervention using the RE-AIM framework. The mean knowledge score used to assess effectiveness was derived from the 15 questions that assessed patients on their knowledge about diabetes and its management. These were multiple-choice questions, adapted from validated tools used by Palaian et al. (28), with correct answers coded as ‘1’ and wrong answers as ‘0’. The value of the mean knowledge score ranged between 0 and 15 and is reported with standard deviation. The mean practice score was derived from the 13 questions that assessed a range of self-management practices, such as seeking diabetes care, regular intake of medication, and lifestyle changes. These questions were adapted from the tool used by Palaian et al. (28) and the WHO STEPS instrument validated for use in India (29). Favorable or ‘healthy’ practices were coded as ‘1’ and all others as ‘0’. The value of the mean practice score ranged between 0 and 13 and is reported with standard deviation. The out-of-pocket expenditure was derived as sum of the expenditures reported by patients on consultation, laboratory tests, food, travel, and any informal payments for a single episode of outpatient care, including medications for 1 month. The mean out-of-pocket expenditure is reported in Indian rupees (INR) with standard deviation. The mean fasting blood sugar was estimated using the blood glucose monitoring device after overnight fasting by patients. The mean fasting blood glucose is reported in mg/dL with standard deviation.

**Table 3:** Measures and source of data for intervention assessment

RE-AIM dimension	Measure	Source of data
Reach	Proportion of the patients who solely visited intervention sites during the intervention period	Post-intervention survey
	Proportion of the patients solely visiting intervention sites who saw posters as well as videos about diabetes at the intervention sites	Post-intervention survey
	Proportion of the patients solely visiting intervention sites who were prescribed generic medications by intervention doctors	Post-intervention survey
	Feedback from the doctors at intervention sites about use/non-use of the standard treatment guidelines for diabetes management	Interviews with doctors
Effectiveness	Mean knowledge scores of patients in the intervention and control groups, before and after the intervention	Pre-intervention survey; post-intervention survey
	Mean practice score of patients in the intervention and control groups, before and after the intervention	Pre-intervention survey; post-intervention survey
	Mean out-of-pocket expenditure by patients in the intervention and control groups, before and after the intervention	Pre-intervention survey, post-intervention survey
	Mean fasting blood sugar of patients in the intervention and control groups, before and after the intervention	Pre-intervention survey; post-intervention survey
Adoption	Adoption of the intervention components by the doctors for implementation at the intervention sites	Interviews with doctors; field observations at the intervention sites
Implementation	Implementation of the intervention as envisaged throughout the 6-month intervention period	Interviews with doctors; field observations at the intervention sites
Maintenance	Delivery of the intervention at intervention sites after 6-month intervention period	Field observations at the intervention sites

RE-AIM: reach, efficacy/effectiveness, adoption, implementation, and maintenance

The data collection tools included a survey of diabetes patients (before and after the intervention), interviews with the intervention doctors at the end of the intervention, and field observations by the first author throughout the intervention, including 3 months past the intervention.

#### Survey of diabetes patients

The questionnaire was field-tested, revised, and translated into local languages. See Supplementary File 1 for a copy of the questionnaire (in English) that sought data about sociodemography, health-seeking, exposure to posters/videos, treatment details, healthcare expenditure, knowledge, and practice in regard to diabetes and perceived social support. The community health workers administered a questionnaire to the sampled patients in a language (Kannada, Urdu, or Tamil) that patients were comfortable with. The community health workers carried out a blood sugar test using a blood glucose monitoring device at the patients’ residence after an overnight fast by the patients. The health workers were trained in questionnaire administration as well as the use of the glucose monitoring device. The same questionnaire and the glucose monitoring technique were used for the initial survey and a follow-up survey after the 6-month intervention period. A trained data entry officer entered the data using EpiData Entry (EpiData Association 3.1). We used STATA (StataCorp LP 11.2) to do difference-in-differences analysis to ascertain the effectiveness of the intervention on the knowledge, self-management practices, out-of-pocket healthcare expenditure, and fasting blood sugar of the patients (see Table 3). This approach takes into consideration the initial differences among intervention and control groups and helps control for unobserved factors that would have affected outcome variables in both the groups. It assumes that the change in both the groups follows a similar trend. We assessed the difference-in-differences estimator adjusted with relevant covariates, including sociodemographic [sex, age, education, income, household poverty status, marital status, religion, and social support score estimated using the Duke social support and stress scale (30)] and disease-related (diabetes duration, comorbidity) variables. The survey data were also used to assess the reach of the intervention.

#### Interviews with doctors

At the end of the intervention period, the first author, with formal training in qualitative research, conducted interviews with the four intervention doctors to understand their experience and views with regard to implementation of the intervention, adaptations that they made to the initial intervention, impact of the intervention on patients, and sustainability of the intervention. The doctors were the respondents of choice, as they owned and managed the intervention sites while also providing healthcare to diabetes patients. A semi-structured questioning guide was developed, pretested, and refined (see Supplementary File 2). Audio-recorded interviews that lasted 45 to 60 min were conducted in a mix of English and Hindi. Open-ended questions were followed with more specific probes to clarify and extend the responses. Records were transcribed verbatim and translated into English by a professional transcriptionist. The first author verified the transcripts based on audio records. Transcripts were analyzed by using thematic content analysis to identify emerging themes. Data were organized and coded using NVivo software (QSR International 8.0) and later the relationship between and across themes was explored using the mind-mapping tool MindNode Lite (IdeasOnCanvas GmbH 1.9.1). Data from the post-intervention survey (about reach of the intervention) and field observations were used for triangulation.

#### Field observations and discussions with the doctors

The first author made follow-up visits to the intervention doctors once a month to discuss the status of the implementation of the intervention and gather their feedback. He made observations about the implementation of the intervention (especially the television/poster component) as well as about the reactions of the patients and health workers. He used a structured observation grid and pointers for discussion with the doctors (see Supplementary File 2). Immediately after the visit, observations and the discussion were recorded in an online field diary, Evernote (Evernote Corporation 2.0.5). These data were analyzed in the same way as the interviews to understand implementation, adoption, and sustainability aspects of the intervention.

## Results

### Participant characteristics

We had four interventions and four control sites (see Table 2). We were able to recruit 317 diabetes patients (163 in the intervention and 154 in the control group) from the community. Table 4 provides sociodemographic and diabetes-related indicators for the sample population at the baseline. Women constituted over two-thirds of the sample population. The sample population had mean per capita income of INR 1,994.3 (approximately USD 32) per month. More than one-fourth (28.7%) of the sample population had no formal education. Of the 33.2% of the sample population who earned income, around 28.6% were daily wage earners. The majority of women were homemakers. The patients in the intervention group had a lower education level and were poorer compared to those in the control group. However, these differences were statistically insignificant.

**Table 4:** Major characteristics of the sample population at baseline

	Control (*N*=154)	Intervention^a^ (*N*=163)
Sex, *n* (%)		
Men	46 (29.9)	52 (31.9)
Women	108 (70.1)	111 (68.1)
Age in years, mean (SD)	52.9 (9.3)	50.8 (9.9)
Education, *n* (%)		
No formal education	40 (26)	51 (31.3)
Up to 5th standard	34 (22.1)	53 (32.5)
Up to 10th standard	64 (41.6)	49 (30.1)
Above 10th standard	16 (10.4)	10 (6.1)
Income per capita per month in INR, mean (SD)	2,095.2 (1,159)	1,906.5 (1,183.6)
Work, *n* (%)		
Employed	16 (10.4)	12 (7.4)
Self-employed	19 (12.4)	28 (17.2)
Daily wage earner	10 (6.5)	20 (12.3)
Unpaid work	4 (2.6)	8 (4.9)
Homemaker	86 (56.4)	74 (45.4)
Retired	8 (5.2)	4 (2.5)
Unemployed	10 (6.5)	17 (10.4)
Household poverty status as per ration card, *n* (%)		
Above the poverty line	120 (77.9)	109 (66.9)
Below the poverty line	8 (5.2)	10 (6.1)
No ration card	23 (14.9)	42 (25.8)
Marital status, *n* (%)		
Currently married	106 (68.8)	111 (68.1)
Separated/divorced	17 (11)	21 (12.9)
Widowed	30 (19.5)	28 (17.2)
Never married	1 (0.7)	3 (1.8)
Religion, *n* (%)		
Hinduism	39 (25.3)	28 (17.2)
Islam	97 (63)	123 (75.5)
Christianity	18 (11.7)	12 (7.4)
Diabetes duration in completed years, mean (SD)	6.6 (0.4)	6.6 (0.4)
Social support score, mean (SD)	55.5 (1.8)	55.2 (1.8)
Knowledge score, mean (SD)	5.8 (2.3)	5.7 (2)
Practice score, mean (SD)	6.6 (0.1)	6.8 (0.1)
Fasting blood sugar in mg/dL, mean (SD)	196.5 (94.5)	212 (94.5)

a The difference between the control and the intervention groups was not statistically significant (at *p*<0.05) when assessed using comparative statistics: t-test and chi-square for comparing means and proportions, respectively. INR: Indian rupees.

### RE-AIM framework

#### Reach of the intervention

**Fig. 1:**
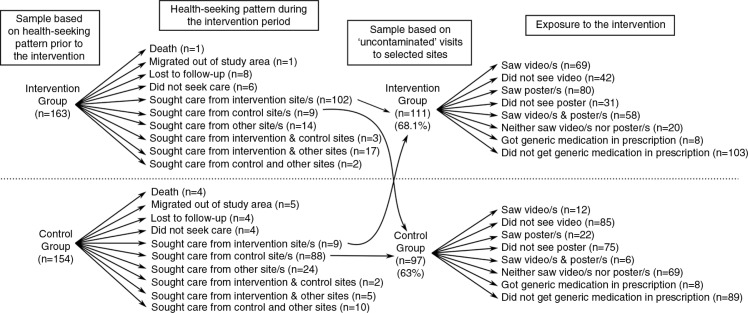
Health-seeking patterns and exposure to the intervention.

Of the patients recruited into the intervention and control groups, 68.1 and 63% visited solely the intervention and control sites, respectively, at least once during the intervention period. Death, migration out of the study area, loss to follow-up, visits to non-selected sites, visits to both the intervention and the control sites by the same patients, and not seeking healthcare at all marked healthcare-seeking for the remaining patients during the 6-month intervention (see Fig. 1). Just over half (52.3%) of those who solely sought care from intervention sites reported seeing posters as well as videos about diabetes. Fewer (7.2%) got generic medications prescribed by the intervention doctors. The doctors at the intervention sites reported a very limited use of STG in their practice. At two of the intervention sites, doctors who practiced modern medicine without the required formal training saw diabetes patients. These doctors found the STG useful and reported using it in their practice. Patients’ exposure to the intervention was not related to their sociodemographic characteristics (sex, age, work, education, income, household poverty status, marital status, religion, social support) or disease condition (diabetes duration, comorbidity), as these variables had no statistically significant association with the probability of patients receiving or not receiving the intervention.

### Effectiveness of the intervention

We compared effectiveness measures between patients in the intervention group who were exposed to the full education component (*n*=58) and those from the control group who had no exposure to the videos and/or posters about diabetes (*n*=69). The size of these groups was smaller than the minimum sample size estimated for the study, compromising the power to detect the expected impact. We did not assess the effectiveness of the generic medication and STG components, as only eight patients had received generic medications in the intervention group and the intervention doctors rarely used STG for diabetes management. The unadjusted difference-in-differences analysis showed that after the intervention the mean knowledge score improved marginally (+0.23) while the mean out-of-pocket expenditure decreased (−29.21). The mean practice score deteriorated marginally (−0.48) and the mean fasting blood sugar level increased (+3.83). These changes were statistically insignificant. After adjusting for sociodemographic and disease-related covariates, the direction of change in effectiveness measures and its statistical insignificance remained same, except that the marginal reduction in the mean practice score (−0.85) became statistically significant (see Table 5).

**Table 5:** Impact of the education component of the intervention

	Intervention (I)	Control (C)	Difference (I–C)	Unadjusted difference-in-differences estimator (SD)	Adjusted difference-in-differences estimator^a^ (SD)
	Mean knowledge score (SD)				
Before (B)	5.36 (0.22)	5.86 (0.27)	−0.49 (0.36)	0.23 (0.51)	0.32 (0.50)
After (A)	6.53 (0.22)	6.80 (0.27)	−0.26 (0.36)		
Difference (A–B)	1.17* (0.31)	0.94* (0.38)			
	Mean practice score (SD)				
Before (B)	7.41 (0.23)	6.90 (0.21)	0.52 (0.31)	−0.48 (0.22)	−0.85* (0.37)
After (A)	6.43 (0.17)	6.39 (0.16)	0.04 (0.23)		
Difference (A–B)	−0.98* (0.29)	−0.51 (0.26)			
	Mean out-of-pocket expenditure				
Before (B)	455.57 (44.65)	471.26 (58.50)	−15.69 (75.82)	−29.21 (187.87)	−65.12 (197.18)
After (A)	965.17 (72.28)	1,010.07 (145.34)	−44.90 (171.89)		
Difference (A–B)	509.60* (84.96)	538.81* (156.67)			
	Mean fasting blood sugar (mg/dL)				
Before (B)	200.26 (10.68)	203.69 (10.09)	−3.43 (14.71)	3.83 (0.84)	7.79 (19.39)
After (A)	206.21 (8.18)	205.82 (8.95)	0.40 (12.40)		
Difference (A–B)	5.95 (13.48)	2.12 (13.45)			

The difference-in-differences estimator was estimated using linear regression.

a Adjusted with covariates (sex, age, work, education, per capital income, household poverty status, marital status, religion, social support score, diabetes duration, comorbidity).

* Two-sided *p*-value<0.05.

### Adoption of the intervention

All the intervention sites adopted the education component of the intervention. All but one site adopted the component of prescribing generic medications. This particular site had its own pharmacy and so preferred to dispense medication brands that they had in stock. The four intervention sites were generally representative of the clinics and hospitals found in the area in terms of infrastructure and services. However, they differed in one important aspect: the doctors at these sites indicated their willingness to make changes in their routine practice. A few additional health facilities in the area (one government and two private) showed willingness to experiment with the intervention, but they could not be part of the study for the reasons cited earlier in methods section.

### Implementation of the intervention

The implementation of the intervention varied across intervention sites as well as across the different components of the intervention.

#### Education component

This component was the most widely implemented; posters and videos were displayed at conspicuous places at all the four sites. One of the sites preferred the installation of the television monitor inside the consultation room, unlike the other three sites, which installed it in the patient waiting areas. This decision was mainly due to the doctor's concern about the safety/security of the monitor, as the doctor worked alone at that facility with no support staff manning the patient waiting area. This change also meant that at this site the intervention was delivered individually and selectively to diabetes patients as part of the consultation process. The researcher's field observations indicated that three of the intervention sites routinely displayed videos during peak consultation hours. However, one of the sites hardly switched the monitor on. It was a busy site, and the responsibility for operating the monitor was vaguely allocated between receptionist and pharmacist with no supervision, leading to almost no use of the monitor during the intervention period. One of the doctors, at the request of some of his patients, provided copies of the video files to the patients.

Three of the videos were marginally edited during the early intervention period when one intervention doctor pointed out that patients were curious about those films referring to data from another country instead of India. Similarly, a poster on dietary information was slightly modified at the request of one the doctors, as there was a mention of pork meat, which could have hurt the sentiments of Muslim patients.

#### Prescription of generic medications

During the intervention period, only eight patients received prescriptions of generic medications. The following themes that emerged from interviews with the doctors explain this poor implementation.

#### Low efficacy and quality

Two of the doctors expressed their doubts about the efficacy of generic medications. They believed that generic medications often come with less strength or lower amounts of the base ingredient compared to what is specified on their packaging. Their concerns were rooted in their past experiences and personal assessments of the use of generic medications.

We are not getting 100% efficacy [with generic medications]. Patients are taking three to four tablets and still their sugar levels remains high, in spite of the diet, physical exercise, and patients’ mental status. The moment we change [to brand-name] medications, we get improvement. (R1, clinic)

Prescription of brand-name medications containing a combination of drug compounds (as opposed to single-compound generics) seems to be a norm, not just for diabetes but also for most other ailments. One of the doctors claimed that the patients in the area have either become ‘resistant’ to single-compound drugs (drawing a parallel with how, over time, bacteria develop resistance to antibiotics) or have become ‘used to’ combination drugs.

See, most of the single [compound] drugs are not working, be it antibiotic or pain killer …. We have to give combinations. (R3, hospital)

When probed about the safety of such a practice, the doctor confided that the safety of such medications has not been ascertained and it could be problematic in the long term. However, the notions that combination drugs give faster results and the primacy accorded to meeting patients’ expectations, at the expense of available scientific knowledge in a competitive commercial healthcare sector, seem to drive this practice. Doctors often doubted the quality of generic medications.

While some generics are good and efficacious, many are produced in small places, like a small house, and are of poor quality. (R3, hospital)

#### Poor acceptability by patients

Generic medications are available as single-drug compounds. Diabetes patients, who often suffered from other morbidities and who were routinely prescribed fewer brand-name medications containing a combination of drug compounds, had to take multiple generic medications.

They cannot take five or six tablets daily. For hypertension, they have to take one tablet. Automatically cholesterol will be there and other problems will be there. (R2, clinic)

#### Limited availability

Considering that a part of the population in KG Halli and the surrounding neighborhoods tend to migrate and keep shifting their residence, the lack of universal availability of generic medications was another concern.

We have a migrating crowd. That is why I don't prefer to use generic medicines, specifically in diabetes and in hypertension kind of prolonged illnesses. (R1, clinic)

#### Use of the standard treatment guidelines

The doctors at the intervention sites reported a very limited use of STG in their practice. Two major themes defined poor use of STG.

#### Patients’ expectations of a doctor

Doctors were expected to be primarily responsible for treating the ailments and treatment was seen as prescription of medications and/or some active intervention. This understanding implies that the doctors found it difficult to promote the active role of patients and the use of non-medical avenues (e.g. self-management practices) in diabetes management.

See, 50% (of diabetes management) is by the doctor and 50% is by patients. But once patients come, it becomes [the] responsibility of doctors to treat them, whether they take care of themselves or not. That's where we are facing the problem. (R4, hospital)

#### Limited role of primary care doctors

The other factor that constrained doctors from using the STG was that they rarely diagnosed new cases of diabetes. They generally did follow-up consultations with patients, who were often diagnosed and put on a given treatment plan by specialists.

The guidelines could be fully practiced if the patient is being newly diagnosed as diabetic. Eighty percent of my patients are already diagnosed with diabetes and I am just following them. (R2, clinic)

### Maintenance of the intervention

The field observations, made for 3 months post-intervention, confirmed the ongoing use of posters and television monitors for health education at the intervention sites. The use of generic medications and STG for diabetes management remained very limited, as was the case during the intervention period.

## Discussion

We found the RE-AIM framework useful in assessing the intervention in its different dimensions and relevant from a public health viewpoint. The health service intervention – aimed at promoting culturally appropriate health education, generic medications, and STG for diabetes management – reached a very limited number of patients, especially with regard to use of generic medications and STG for diabetes management. It did not have a statistically and clinically significant impact on the knowledge, out-of-pocket healthcare expenditure, or glycemic control of patients with an (albeit marginal) reduction in their mean practice score. The absence of any impact can be explained by poor implementation of the intervention, reflecting non-acceptance and/or lack of willingness of doctors to change at the intervention sites. Doctors’ concerns about the efficacy, quality, availability, and acceptability by patients of generic medications explained the limited prescriptions of generic medications. The patients’ perception that ailments should be treated through medications limited the use of non-medical management by the doctors for the early stages of diabetes. The other reason for the limited use of the standard treatment guidelines was that these doctors mainly provided follow-up care to patients who were previously put on a given treatment plan by specialists. Positively, the doctors perceived that the culturally relevant education delivered in local languages and videos generated curiosity among patients, who felt more confident in asking questions, leading to enhanced knowledge and self-management practices.

We identified the reasons why the intervention, which was delivered in a real-world, resource-constrained setting, was not found to be effective. First, merely making low-cost generic medications available was not sufficient to reduce treatment cost for patients. The doctors’ perceived low efficacy and availability as well as acceptability by patients of generic medications were reasons for their poor use. Concern about the quality of generic medications is widespread among doctors beyond KG Halli and to some extent seems justified. Ravinetto et al. (31) highlight how the poor quality of some generic medications and the resultant poor perceptions of Indian generics negatively impact equitable access to healthcare not just for communities in India but worldwide, as India remains a huge supplier of generic medications to many low-income countries. Along the same lines as the committee set up by the Indian Parliament (32), Ravinetto et al. (31) point to the need for more transparency and effective regulation of the pharmaceutical sector. In addition, the poor adherence to the practice of prescribing generic medications could also be explained by Indian law, as pharmacists are legally not allowed to replace prescribed brand medication with generic counterparts. Our earlier work in KG Halli highlighted the practice of kickbacks by private pharmacies to doctors for prescribing brand-name medications (4). Policies, including the proposed free drug scheme of the government of India (33), that aim to improve access to affordable medications need to address the various factors outlined, beyond making medications available at low cost or for free.

The very limited use of STG for diabetes management was explained by the fear of medical doctors that lack of prescription is perceived as lack of treatment by the patients. Studies from Australia (34) and the United Kingdom (35–38) reveal that patients’ expectations with respect to prescriptions – and even more strongly, the doctors’ perceptions of patients’ expectations – are important factors impelling doctors to prescribe unnecessary medications. In a study in New Delhi (India), the doctors indicated that their patients’ demands and expectations for antibiotic prescriptions was an important factor influencing their prescriptions for antibiotics (39). In India, there is a dearth of research exploring patients’ expectations of their doctor, doctors’ perceptions of what their patients expect from them, and the interactions between the two. Such studies would help in better understanding the patient–provider relationship and how that in turn influences management of diabetes and other chronic conditions.

The limited role of the primary care doctors in deciding the treatment plan for diabetes patients was another reason for poor use of STG. This highlights the fragmentation of healthcare services, with poor referral links across the types and levels of healthcare services in India. This situation makes it difficult to coordinate and ensure continuity in patients’ care. Our earlier analysis (4) of the local health system in KG Halli points to this systemic impediment, which is in sharp contrast to the model (40) where primary care is at the very center of the health system, serving as a hub of coordination with the different actors in the community and at the various levels of healthcare and social services.

Our study has limitations. Due to limited resources, we opted for a shorter intervention period of 6 months. A longer intervention duration would have helped overcome some of the implementation challenges identified during the course of the intervention. Our study also suffered from high crossover and contamination, where many patients either visited sites other than the study sites or they visited both the intervention and control sites, reducing the uncontaminated sample beyond the minimum sample size needed to detect the predicted change in outcome variables. Although we envisaged and accounted for this in the sample size calculation, the extent of contamination was greater than we expected. The use of the RE-AIM framework, however, helped us to understand other important dimensions of the intervention, beyond the effectiveness, including the reasons for poor implementation. Our findings provide valuable insights to public health authorities about some of the challenges and opportunities for reforming healthcare services to improve care for diabetes and other chronic conditions, especially in harnessing the huge private health sector in India (3, 41).

## Conclusions

This health service intervention – aimed at promoting culturally appropriate health education, generic medications, and STG for diabetes management – reached a very limited number of patients. It did not have statistically or clinically significant impact on the knowledge, out-of-pocket healthcare expenditure, or glycemic control of patients with an (albeit marginal) reduction in their mean practice score. The doctors, however, perceived that the culturally relevant education delivered in local languages and the videos generated curiosity among patients, who felt more confident in asking questions leading to enhanced knowledge and self-management practices. Implementing an efficacious health service intervention in a real-world resource-constrained setting is challenging and may not prove effective in improving patient outcomes. Interventions need to consider patients’ and healthcare providers’ experiences and perceptions and how macro-level policies translate into practice within local health systems.

## Supplementary Material

Intervening in the local health system to improve diabetes care: lessons from a health service experiment in a poor urban neighborhood in India

Intervening in the local health system to improve diabetes care: lessons from a health service experiment in a poor urban neighborhood in India
