# Supplementary material for: Intervening in the local health system to improve diabetes care: lessons from a health service experiment in a poor urban neighborhood in India
Source: Glob Health Action. 2015 Nov 16;8:10.3402/gha.v8.28762. doi: 10.3402/gha.v8.28762 (PMC4649018; doi:10.3402/gha.v8.28762)
Supplement: Intervening in the local health system to improve diabetes care: lessons from a health service experiment in a poor urban neighborhood in India [file GHA-8-28762-s001.pdf]

Questionnaire for diabetes patient to be administered by Community Health Worker

Consent & Interview Details

1	Consent has been read and obtained	1. Yes<br>2. No (end the interview) ☐
2	Name of the interviewer	
3	Date of the interview (DD/MM/YYYY)	/ /2013
4	Name of the respondent	
5	Household address of respondent: Door number	
	Street name	
	Area/slum name	
	Landmark	
6	Contact phone number	
7	Participant ID (to be generated later by researcher)	

Demographic information

8	What is your age (in completed years)?	
9	Sex	1. Male<br>2. Female<br>3. Transgender ☐
10	Which language do you commonly speak in household?	1. Kannada<br>2. Urdu<br>3. Tamil<br>4. Malayalam<br>5. Telugu<br>6. Hindi<br>7. English<br>8. Other (Specify):________________ ☐
11	What is the highest level of education you have completed?	1. No formal schooling<br>2. Less than primary school (< 5th class)<br>3. Lower Primary school completed (till 5th class)<br>4. Upper Primary school completed (till 7th class)<br>5. Secondary school completed (till 10th class)<br>6. Pre University/Higher Secondary school completed (till 12th class)<br>7. Graduation (University degree/diploma)<br>8. Post-graduation<br>99. Refused ☐
12	Which language/s you could read and understand? (Select as many as applicable)	Read ☐☐☐ Understand ☐☐☐<br>1. Kannada<br>2. Urdu<br>3. Tamil<br>4. Malayalam<br>5. Telugu<br>6. Hindi<br>7. English<br>8. Other (Specify):________

13	Religion	1. Hinduism<br>2. Islam<br>3. Christianity<br>4. Others (specify):___________ ☐
14	Caste	1. General category<br>2. Schedule Caste (SC)<br>3. Schedule Tribe (ST)<br>4. Other Backward Caste (OBC)<br>98. Do not know<br>99. Refused ☐
15	Marital status	1. Never married<br>2. Currently married<br>3. Separated<br>4. Divorced<br>5. Widowed<br>99. Refused ☐
16	What describes best your main work status over the last six months?	1. Government employee<br>2. Private (formal sector) employee<br>3. Private (informal sector) employee<br>4. Daily wage earner<br>5. Self-employed<br>6. Unpaid work<br>7. Student<br>8. Homemaker<br>9. Retired<br>10. Unemployed<br>99. Refused ☐
17	Number of people, including you, living in this household	
18	Number of people, including you, who are above 18 years	
19	Number of people, including you, who are currently earning members	
20	What is your relation with the head of the household?	1. Self<br>2. Father<br>3. Mother<br>4. Husband<br>5. Wife<br>6. Son<br>7. Daughter<br>8. Brother<br>9. Sister<br>10. Daughter in law<br>11. Other (Specify):__________________ ☐
21	Taking the past year, can you tell me what has been the average monthly earning of the household?	Average Monthly earnings (in INR) ☐<br>98. Don't know<br>99. Refused
22	Availability and type of ration card	1. No ration card<br>2. Above Poverty Line card<br>3. Below Poverty Line card<br>4. Antyodaya card<br>99. Refused ☐

Self-reported health and health-care experiences

23	How long ago you came to know about your diabetes (number in completed years)					
24	Do you visit a doctor for diabetes care at least once every three months?					1. Yes ☐<br>2. No
25	In last six months, how many times you visited a doctor for diabetes?					
26	Which doctor/s you visited for diabetes in last six months?	Name of facility & area<br>1st Visit……<br>2nd Visit……<br>3rd Visit……<br>4th Visit……<br>5th Visit……<br>6th Visit……				
Try to remember the visits to clinics/hospitals you made in last six months for diabetes care and answer the following questions for each of the visit. Answer codes: 1=Yes, 2=No, 98=Don't know						
		1st Visit	2nd Visit	3rd Visit	4th Visit	5th Visit
27	Did you see any poster on diabetes in a language that you could easily read?	☐	☐	☐	☐	☐
28	Did you see any video on diabetes in a language that you could easily understand?	☐	☐	☐	☐	☐
29	Did your doctor discuss with you about diabetes? (Advise/suggestion beyond the medical prescription)	☐	☐	☐	☐	☐
30	What treatment did your doctor prescribe to you for diabetes in your last visit to a doctor? Ask for a prescription and note down names/dosage/frequency of medications prescribed. If prescription is not available, ask for medications and try to note down the details on medications.					

(A) Prescri-ption available (1=Yes2 =No)	(B) Form (e.g. Tab.)	(C) Name of medication	(D) Dosage (e.g. 10 mg)	(E) Frequency of daily intake (2 times/day)	(F) Duration (e.g. 7 day)	(G) Patient's practice (Use following codes, use as many as applicable)<br>1. Taking regularly as prescribed<br>2. Missing out on medication<br>3. Not taking medications<br>4. Others (specify)

31	Do you currently have any illness, lasting for more than 30 days, apart from diabetes? (Select as many as applicable). Mention the duration of illness (in years) against the name of the illness.	1. None<br>2. Hypertension (BP)<br>3. Heart problem (specify):_______<br>4. Asthma<br>5. Stroke<br>6. Kidney problem (specify):_________<br>7. Eye problem (specify):_____________<br>8. Thyroid problem (specify):____________<br>9. Fits (seizures)<br>10. Body pain (specify):________________<br>11. Nerve problem<br>12. Others (Specify):_______________
32	Does any other household member/s has/have any illness lasting for more than 30 days? (Select as many as applicable).	1. None<br>2. Diabetes (Sugar)<br>3. Hypertension (BP)<br>4. Heart problem (specify):_______<br>5. Asthma<br>6. Stroke<br>7. Kidney problem (specify):_________<br>8. Eye problem (specify):_____________<br>9. Thyroid problem (specify):____________<br>10. Fits (seizures)<br>11. Body pain (specify):________________<br>12. Others (Specify):_______________

		Total	Only Diabetes
33	Taking the last six months, can you tell me what has been the average **monthly** spending **on medications**?		
34	Taking the last six months, can you tell me how much **consultation fee** did you pay to doctor in a **single visit**?		
35	Taking the last six months, can you tell me what has been the average spending **on laboratory tests** for a **single visit** to a doctor?		
36	Can you tell me what has been the average spending **on travel** for a **single episode** of care-seeking (visit to doctor, laboratory and pharmacy)		
37	Do you need to make **informal payments (speed money or bribe)** while seeking care for diabetes? If No, put zero in the box. If Yes, how much money you have to generally spend on informal payments for a **single episode** of care?		
38	Do you need to buy food from outside while you visit doctor, pharmacy or laboratory for diabetes care? If No, put zero in the box. If Yes, how much do you spend **on food** for a **single episode** of care?		
39	Do you **miss out on your earnings** (wage loss) while you visit doctor, pharmacy of laboratory for diabetes care? If No, put zero in the box. If Yes, how much earning you loose out like this for a **single episode** of care?		

40	What was the source of money that you spent on treatment (Select as many as applicable)?	1. From pocket<br>2. Dip into savings<br>3. Borrowed money without interest<br>4. Borrowed money with interest<br>5. Mortgaged assets<br>6. Sold assets<br>7. Others (Specify)<br>98. Do not know

Knowledge about Diabetes

41	Diabetes is a condition in which the body contains…	1. A higher level of sugar in the blood than normal<br>2. A lower level of sugar in the blood than normal<br>3. Either a higher or a lower level of sugar in the blood than normal<br>98. Don't know
42	Major cause of diabetes is…	1. An increased availability of insulin in the body<br>2. A decreased availability of insulin in the body<br>98. Don't know
43	The symptoms of diabetes are…	1. Increased frequency of urination<br>2. Increased thirst and hunger<br>3. Increased tiredness<br>4. Slow healing of wounds<br>5. All of the above<br>6. Some of the above<br>7. None of the above<br>98. Don't know
44	Diabetes, if not treated…	1. Can lead to eye problems<br>2. Can lead to kidney problems<br>3. Can lead to foot ulcers<br>4. Can lead to heart problem<br>5. All of the above<br>6. Some of the above<br>7. None of the above<br>98. Don't know
45	Best (most accurate) method of monitoring diabetes is…	1. Blood testing<br>2. Urine testing<br>3. Both are equally good<br>98. Don't know
46	The important factors that help in controlling (blood) sugar are…	1. Controlled and planned diet<br>2. Regular exercise<br>3. Regular medication<br>4. All of the above<br>5. Some of the above<br>6. None of the above<br>98. Don't know
47	Upon control of diabetes, medications…	1. Can be stopped immediately<br>2. Can be stopped after a month<br>3. Should be continued for life<br>98. Don't know

48	Can (blood) sugar become low while you are taking diabetes medicines?	1. Yes<br>2. No<br>98. Don't know	☐
49	What happens when the blood sugar becomes low?	1. Sweating<br>2. Dizziness/giddiness<br>3. Blurred vision<br>4. Tiredness /Weakness<br>5. All of the above<br>6. Some of the above<br>7. None of the above<br>98. Don't know	☐
50	What should be done when blood sugar becomes low?	1. Eat sugar, candy or something with sugar<br>2. Take medicines<br>3. Take insulin<br>98. Don't know	☐
51	Numbness and tingling could be symptoms of…	1. Kidney problems<br>2. Eye problems<br>3. Nerve problems<br>4. Liver problems<br>98. Don't know	☐
52	The best way to take care of your feet is to…	1. Look (inspect) at and wash them daily<br>2. Walk barefoot inside and outside the house<br>3. Buy a shoes a size larger than usual<br>98. Don't know	☐
53	Which of these items could be freely eaten without any restrictions…	1. Chicken<br>2. Beans<br>*3. Jaggery*<br>4. All of the above<br>5. Some of the above<br>6. None of the above<br>98. Don't know	☐
54	Which of the fruits should be avoided…	1. Pomegranate<br>2. Papaya<br>3. Banana<br>4. All of the above<br>5. Some of the above<br>6. None of the above<br>98. Don't know	☐
55	Lifestyle changes required for diabetes patients are…	1. Weight control/reduction<br>2. Stopping smoking<br>3. Stopping alcohol intake<br>4. All of the above<br>5. Some of the above<br>6. None of the above<br>98. Don't know	☐

Practice in regard to diabetes (in addition to what is not covered earlier)

56	Do you currently smoke any tobacco products (e.g. cigarette, *bidi* etc.)	1. Yes<br>2. No<br>If NO, go to Q.59	☐

57	Do you currently smoke any tobacco products **daily**?	1. Yes<br>2. No ☐
58	On an average how many of these smoking products do you smoke each day?	Cigarettes:<br>*Bidis:*<br>Others (specify):……..
59	Do you currently use any smokeless tobacco products (such as *Gutka, Khaini, Bida* with tobacco etc.)?	1. Yes<br>2. No ☐<br>If NO, go to Q.62
60	Do you currently use any smokeless tobacco products **daily**?	1. Yes<br>2. No ☐
61	On an average, how many times a day do you use these products?	*Gutka*……………………<br>*Khaini*……………………..<br>*Bida* with tobacco……<br>Other (specify)…………
62	Have you consumed an alcoholic drink within the past 30 days?	1. Yes<br>2. No ☐
63	During the past 30 days, on how many occasions did you have at least one alcoholic drink?	
64	Do you do vigorous-intensity activity (as part of work, household chores, travel or leisure) that requires hard physical efforts causing large increases in breathing or heart rate [like carrying or lifting heavy loads, digging or construction work, running etc.] for at least 10 minutes continuously?	1. Yes<br>2. No ☐<br>If NO, go to Q.67
65	In a typical week, on how many days do you do such vigorous-intensity activities?	
66	How much time do you spend doing such vigorous-intensity activities at on a typical day?	
67	Do you do moderate- intensity activity (as part of work, household chores, travel or leisure) that requires moderate physical efforts causing small increases in breathing or heart rate like [brisk walking, carrying or lifting light loads, cycling, swimming, doing manual household chores like mopping, sweeping etc.] for at least 10 minutes continuously	1. Yes<br>2. No ☐<br>If NO, go to Q.70
68	In a typical week, on how many days do you do such moderate-intensity activities?	
69	How much time do you spend doing such moderate-intensity activities at on a typical day?	
70	In a typical day, how many times a day you eat fruits and/or vegetables?	1. Less than three times<br>2. Three times or more ☐
71	Are you currently following a controlled and planned diet?	1. Yes<br>2. No ☐
72	When was your blood pressure checked last?	1. One week ago<br>2. One month ago ☐<br>3. Two months ago<br>4. Six months ago

73	When did you have your last eye examination?	1. One month ago<br>2. Six months ago<br>3. One year ago<br>4. Two years ago<br>5. Not done at all	☐
74	When was your last urine examination?	1. One month ago<br>2. Six months ago<br>3. One year ago<br>4. Not done at all	☐
75	Do you take care of your feet on a regular basis? (Washing and inspecting feet)	1. Yes<br>2. No	☐

Patients' perceived quality of care and satisfaction

Considering the your last visit to the doctor for diabetes care, please rate the following questions on a scale of 1-5, where 1=strongly disagree, 2=Disagree, 3=Neutral, 4=Agree, 5=Strongly agree		
76	The doctor/s gave you advise about ways to avoid illness (diabetes) and stay healthy	
77	The doctor gave you complete information about your illness (diabetes)	
78	The doctor gave you complete information about your treatment (diabetes)	
79	Clinic/hospital staff talk politely	
80	Clinic/hospital staff are helpful to you	
81	You are given enough time to tell the doctor everything	
82	Doctor listen carefully to what you have to say	
83	The doctor checks patients properly	
84	The doctor is always ready to answer your questions	
85	The doctor gave you adequate time	

Patients' perceived social support

A supportive person is one who is helpful, who will listen to you, or who will back you up when you are in trouble. Please let me know how supportive the following people are to you at this time in your life. For the answers, use codes: 0=None, 1=Some, 2=A lot, 0=There is no such person		
86	Your wife, husband, or significant other person	
87	Your children or grand children	
88	Your parents or grand parents	
89	Your brothers or sisters	
90	Your other blood relatives	
91	Your relatives by marriage (in-laws etc.)	
92	Your neighbors	
93	Your co-workers	
94	Your church/temple/mosque (faith-based group) members	
95	Your other friends	
96	Do you have one particular person whom you trust and to whom you can go with personal difficulties?	Yes=2<br>No=0 ☐

Blood sugar estimation

97	Consent has been read and obtained	1. Yes<br>2. No (end the interview)	☐
98	Reading of fasting blood sugar level (in mg/dL) by Glucometer		
