# Supplementary material for: Intervening in the local health system to improve diabetes care: lessons from a health service experiment in a poor urban neighborhood in India
Source: Glob Health Action. 2015 Nov 16;8:10.3402/gha.v8.28762. doi: 10.3402/gha.v8.28762 (PMC4649018; doi:10.3402/gha.v8.28762)
Supplement: Intervening in the local health system to improve diabetes care: lessons from a health service experiment in a poor urban neighborhood in India [file GHA-8-28762-s002.pdf]

Semi-structured guide for conducting interviews with intervention doctors at the end of the intervention period

The following questions are to broadly guide the interviews with the intervention doctors. More specific probes can be used for specific interviews based on researchers' observations and discussions with the intervention doctors during the intervention period.

How has been your experience in delivering the intervention in last six-months?
Was it possible to implement the intervention as desired? What kind of challenges you faced in implementation?

How did the intervention evolve over time?
How did you respond to challenges in implementation? What changes you brought to the intervention? What factors affected your decision to change/modify the intervention?

How diabetes (and other) patients reacted to intervention?
Did patients mention/talk with you or your staff about the intervention? What were their reactions/suggestions?

Perceived effects of intervention on diabetes patients
Do you feel the intervention made any difference to diabetes patients? How? (e.g. questions to the doctor, knowledge, participation in decision-making, self-management practices, and overall disease control).

How did the intervention affect other patients and/or healthcare practice in general?
In what ways, implementation of the intervention (for diabetes care) in your clinic/hospital affected your general practice? (Consultation time, active participation of patients by asking questions, discussing limitations etc., demands for health education in general, demands for low-cost medications for other conditions etc.)

What do you think about such intervention in general? Would you like to continue with this? Would you like to do similar things for other diseases/conditions?

Observation grid

On a visit to the intervention site (health facility), at least once in a month, the principal researcher would spend time in the patient waiting area as non-participant observer. The consider points shall guide the observations.

1. Digital display monitor

Is it located at a conspicuous place? How easy it is for the patients and their attendants to watch videos while sitting in waiting area? Is display monitor on? How are patients reacting to videos (watching? Talking about it?)? Were diabetes videos played during the visit? Has the intervention doctor modified or added videos in the display monitor? Any other remarks…

2. Posters

Are posters being displayed in the patient waiting area? Any changes in the display locations/content over time? How are patients reacting to posters? (Noticing? Reading? Discussing?) Any other remarks…

3. Consultation length

Notice and measure the average consultation time that the intervention doctors spend with the patients (preferably diabetes patients).

4. Interactions with patients/healthcare workers

Whenever the opportunity arises, initiate informal conversation with patients (preferably diabetes or chronic condition patients) and healthcare workers to gather their experience/views on poster/videos.

Periodic discussions with the intervention doctors

Apart from the interview at the end of the intervention period, the principal researcher would meet intervention doctors at least once in a month at their clinics/hospitals. In such visits, researcher would initiate informal discussion with the doctors on implementation of intervention and gather their feedback. These discussions with the doctors during monthly visits will be guided by the first five broad themes mentioned in earlier section (semi-structured guide for the interviews).
